# Release and MMP-9 Inhibition Assessment of Dental Adhesive Modified with EGCG-Encapsulated Halloysite Nanotubes

**DOI:** 10.3390/nano13060999

**Published:** 2023-03-09

**Authors:** Saleh Alhijji, Jeffrey A. Platt, Abdulaziz Alhotan, Nawaf Labban, Marco C. Bottino, L. Jack Windsor

**Affiliations:** 1 Department of Dental Health, College of Applied Medical Sciences, King Saud University, Riyadh 11545, Saudi Arabia; 2 Department of Biomedical Sciences and Comprehensive Care, Indiana University School of Dentistry, Indianapolis, IN 46202, USA; 3 Department of Prosthetic Dental Sciences, College of Dentistry, King Saud University, Riyadh 11545, Saudi Arabia; 4 Department of Cariology, Restorative Sciences and Endodontics, School of Dentistry, University of Michigan, Ann Arbor, MI 48109, USA; 5 Department of Biomedical Engineering, College of Engineering, University of Michigan, Ann Arbor, MI 48109, USA

**Keywords:** dentin adhesive, MMP inhibitors, nanotube encapsulation, dentin–resin interface, drug release

## Abstract

Degradation of the collagen fibrils at the dentin–resin interface by the enzymatic activity of matrix metalloproteinases (MMPs) has been known to permit some dental restoration complications, such as microleakage, secondary caries, and, ultimately, restoration failures. This study aimed to evaluate a modified adhesive by adding an MMP inhibitor from green tea extract with and without nanotube encapsulation to sustain the drug release. Epigallocatechin-3-gallate (EGCG) and Halloysite nanotubes (HNTs) were prepared to produce three variant combinations of modified adhesive (EGCG, EGCG-encapsulated HNT, and EGCG-free HNT). The drug loading efficiency and EGCG release over time were evaluated using UV-vis spectrometry. MMP-mediated β-casein (BCN) cleavage rate assays were used to determine the ability of the EGCG in eluates of the adhesive to inhibit MMP-9 activities. For up to 8 weeks, HNT encapsulation reduced release to a statistically significant level. MMP-mediated β-casein cleavage rate assays showed a significant decrease for the EGCG groups compared to the non-EGCG adhesive groups. Furthermore, the use of HNT for EGCG encapsulation to modify a dental adhesive helped slow down the rate of EGCG release without impacting its MMP inhibitory capabilities, which may help to maintain the dentin–resin interface’s integrity over the long term after dental restoration placement.

## 1. Introduction

The intermittent failure of the interfacial integration between the dental resin and the demineralized dentin at the hybrid layer has been identified as a critical element in the failure of dental restorations [1]. Matrix metalloproteinases (MMPs) have been associated with the degradation of the hybrid layer by cleaving collagen fibrils at the interface [2,3], even in the absence of bacteria [4], creating a pathway for destruction by secondary caries and, ultimately, causing restoration debonding that requires additional treatments [5,6]. Furthermore, replacing restorations usually comes at a cost and is associated with undesirable side effects, such as increasing the cavity size, destroying the tooth, or entailing the need for endodontic treatment or even extraction [7,8].

Generally, the activation of MMPs to cleave collagen follows a stepwise cascade. It can be initiated by a protease attacking the exposed region of the MMPs (bait region) between the first and second helices of the propeptide, leading to the destabilization of the rest of the propeptide, including the cysteine-zinc switch [9]. Besides endogenous proteases, MMPs can be triggered by exogenic chemical agents such as oxidized glutathione, sodium dodecyl sulfate (SDS), reactive oxygen species, or abnormal physiological changes [10,11]. In addition, studies have shown that the acidity of the etchant may activate dentinal MMPs [12,13]. However, the mechanism and timing of MMPs involved in the degradation of the dentin–resin interface remain uncertain. Therefore, incorporating MMP inhibitors within dental adhesives has been suggested as a promising therapeutic technique that may prolong the durability of the dentin–resin interface [14].

Epigallocatechin-3-gallate (EGCG) from green tea extract is commonly studied and suggested as a health supplement because of its antioxidant, antimicrobial, antidiabetic, anti-inflammatory, and cancer prevention characteristics [15,16]. Furthermore, researchers have found that EGCG can deactivate MMP and cysteine cathepsin enzymes [17,18,19,20] as well as inhibit the growth and biofilm formation of cariogenic bacteria [16]. The combination of MMP inhibition and antibacterial characteristics makes EGCG an ideal candidate for incorporation in dental adhesives. Several studies have evaluated EGCG addition to dental adhesives to improve the durability of bonding to dentin [21,22,23,24,25,26]. Nevertheless, a lack of understanding remains regarding how quickly a modified adhesive can release EGCG and whether it retains its effectiveness in inhibiting MMPs after incorporation and release processes.

Utilizing nanotubes for drug encapsulation to sustain drug delivery at the dentin–resin interface is considered a novel approach that can be advantageous in sustaining EGCG release. Halloysite nanotube (HNT), which is an aluminosilicate clay mineral (Al_2_Si_2_O_5_(OH)_4_), has been incorporated into adhesives to sustain the release of therapeutic compounds [27]. HNT was identified to have a bilayer roll structure in a cylindrical shape with an axis ratio of 20:1. The space between the layers and central lumen can hold different functional chemicals for slow and sustained release (i.e., hours, days, and months) [28].

The involvement of HNTs as reservoirs to sustain the EGCG release at the dentin–resin interface has not yet been investigated. This study aimed to bridge this gap by evaluating the incorporation of EGCG-encapsulated HNTs into a dental adhesive. The underlying motivation of the study was to maintain the integrity of the dentin–resin interface through effective MMP inhibition, which may contribute to extending the life span of restorations and reducing the failure frequency of dentin bonding. Therefore, we evaluated EGCG release from modified dental adhesives fabricated with and without HNT encapsulation and assessed the bioactivity in terms of MMP inhibition of the EGCG after release.

## 2. Materials and Methods

### 2.1. EGCG Encapsulation

Epigallocatechin-3-gallate (EGCG 95% pure extract; Sigma-Aldrich, St Louis, MO, USA) was dissolved in ethanol to produce a 43.6 mM (20 mg/mL) solution. Two grams of pre-sieved (≤45 µm) halloysite nanotubes (HNTs; Dragonite HP, Applied Minerals Inc., New York, NY, USA) were added to the EGCG solution and vortexed for five minutes before being stored overnight on a rack rotor. The solution containing EGCG-HNT with a final ratio of 10% *w*/*w* was centrifuged at 3000 rpm for 30 min. Vacuum pressure was applied twice, once before and once after the final stirring, and maintained for 30 min (at 25 in Hg) to remove any air pockets. The supernatant was collected to analyze drug loading and speed up the drying process. Finally, the mixture was evaporated in an incubator at 37 °C to obtain a dried powder of EGCG-encapsulated HNTs.

### 2.2. Determining Drug Encapsulation Efficiency

The encapsulation efficiency of halloysite nanotubes (HNTs) for holding EGCG particles was evaluated using direct and indirect methods. The direct technique (*EEd*%) involved quantifying the loaded EGCG amount by dissolving 10 mg of EGCG-encapsulated HNTs in 10 mL of ethanol (EtOH). The EtOH solution was replaced after each reading until no further EGCG particles from HNTs were detected in the solution. The indirect technique (*EEi*%) involved quantifying the unloaded EGCG particles in the supernatant after the encapsulation process. A UV/Visible spectrophotometer (Ultrospec 3100 pro, GE Healthcare, Wallingford, CT, USA) was used to detect the absorption maxima of EGCG at 275 nm through a 1 cm quartz cuvette [29]. Equations *A* and *B* below were used to determine the ratio of the loaded EGCG and to estimate the average encapsulation efficiency (*EE_avg_*%):

(1) $$\left(A\right):EEd\%=\frac{total\;EGCG\;detected\;from\;dissolving\;encapsulated\;EGCG-HNT\;}{total\;EGCG\;added\;}\times 100$$

(2) $$\left(B\right):\;EEi\%=\frac{total\;EGCG\;added\;–\;EGCG\;detected\;in\;the\;supernatnat\;}{total\;EGCG\;added\;}\times 100$$

(3) $$\left(C\right):\;EE_{avg}\%=\frac{EEd\%+EEi\%\;}{2\;}$$

### 2.3. Adhesive Specimen Preparation

A commercially available etch-and-rinse adhesive (Adper Scotchbond Multi-Purpose, 3M Oral Care, St. Paul, MN, USA) was mixed with 7.5% (*w*/*v*) of either EGCG-encapsulated HNTs or HNTs alone. An additional EGCG adhesive group without any HNT encapsulation was included by directly adding 0.15% (*w*/*v*) of EGCG to the adhesive, corresponding to the estimated amounts of EGCG loaded in the EGCG-encapsulated HNT adhesive group. The experimental adhesive groups with the ratios of the components are summarized in Table 1.

The modified adhesive groups were mixed overnight in a dark room at 22 °C using a magnetic stirrer. Teflon molds were used to fabricate disk-like specimens (8.0 × 0.9 mm). Curing was performed using a blue-violet LED curing unit (Bluephase Style; Ivoclar Vivadent, Amherst, NY, USA) with an appropriate output intensity (≥850 mW/cm^2^) monitored between samples using a visible curing light meter (Cure-Rite; LD Caulk/Dentsply, York, PA, USA). Each side of the adhesive specimen was cured for 20 s and then stored at 37 °C in a dark and dry environment for 24 h before the experiments commenced.

### 2.4. EGCG Release Analysis Using UV-Vis

Cured adhesive specimens containing EGCG with and without HNT encapsulation (G3 and G4) were immersed in 2 mL of ultra-pure water (pH = 6.5, *n* = 3/group). The solutions containing EGCG eluates were collected on days 1, 7, 14, 28, and 56 and replaced with fresh ultra-pure water. The wavelength absorbance at 275 nm corresponding to EGCG was quantified using a UV/visible spectrophotometer (Ultrospec 3100 pro, GE Healthcare, Wallingford, CT, USA) through a 1 cm quartz cuvette. The UV-vis calibration curve of EGCG-standardized solutions was used to calculate the EGCG in the eluates.

### 2.5. MMP-Mediated β-Casein Cleavage Assays

A fresh set of cured adhesive specimens (*n* = 3) from each adhesive group was immersed in 2 mL of 50 mM Tris-HCl buffer containing 0.2 M NaCl, 5 mM CaCl_2_, and 1 μM ZnCl_2_ (pH 7.5) and then stored at 37 °C in sealed glass vials. The solutions were collected after 28 and 56 days and stored at −20 °C until testing. β-casein (bCN) was used as a substrate for cleavage rate assessments to determine the inhibitory effects of EGCG-containing eluates against MMP-9 [30]. Briefly, a pre-activated human MMP-9 (0.1 μg/mL) (Sigma-Aldrich) was added to β-casein (12.5 mg/mL) with the eluates from G1, G2, G3, and G4 and incubated at 30 °C. Two additional groups containing blank buffer (no treatment) and 17.5 μg of EGCG (44 μg/mL) were used as negative and positive controls. Samples of 35 μL were periodically (at 0, 15, 30, 60, and 120 min) collected in tubes containing 10% *v*/*v* of 1,10-phenanthroline (36 mg/mL) to terminate the bCN cleavage. The samples were resolved in 15% sodium dodecyl sulfate-polyacrylamide gel electrophoresis (SDS-PAGE) (2.84 mL H_2_O, 2 mL 2M Tris pH 8.8, 5 mL 30% acrylamide, 0.1 mL 10% SDS, 0.05 mL 10% APS, and 0.02 mL TEMED). The gels were stained with Coomassie blue (0.5 g Coomassie brilliant blue R-250, 100 mL glacial acetic acid, 400 mL MeOH, and 500 mL H_2_O), and the rate of disappearance of the 29 kDa of the β-casein (bCN) band was quantified and analyzed using ImageJ software (NIH, Bethesda, MD, Montgomery).

### 2.6. Statistical Analysis

All the data were statistically analyzed using IBM-SPSS software with a 0.05 level of significance. An independent samples t-test was used in drug loading and release analysis to examine the influence of HNTs on the EGCG release. One-way analysis of variance (ANOVA) and Tukey’s multiple comparison tests were performed to identify any statistical difference among the groups in the MMP-mediated β-casein cleavage assay.

## 3. Results

### 3.1. Drug Loading and Unloading Analysis

**Figure 1:**
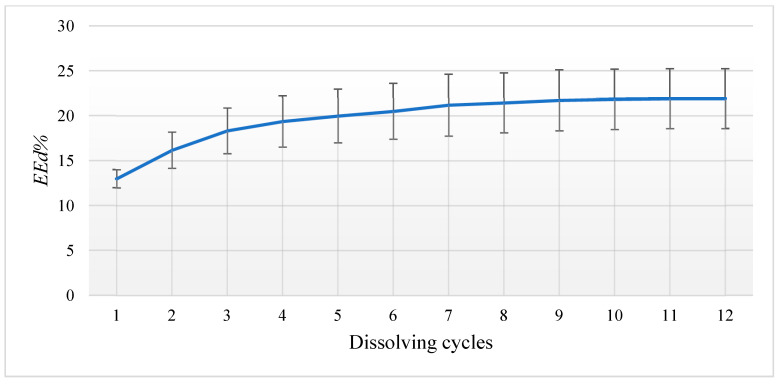
EGCG encapsulation efficiency from repeated dissolving of EGCG-HNT in ethanol.

**Figure 2:**
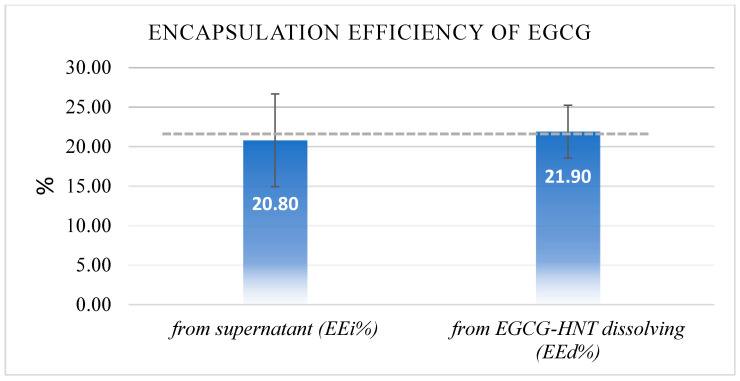
Encapsulation efficiency results of the EGCG detected in the supernatant (unloaded) and dissolved EGCG-encapsulated HNTs (loaded).

The direct detection technique (*EEd*%) by re-dissolving 10 mg of EGCG-HNT (10% *w*/*w*) into 10 mL of ethanol indicated that 21.9% (±3.3%) of EGCG was successfully loaded. The maximum number of dissolving cycles ranged from 10 to 12 cycles before a plateau was reached, which indicated that no further EGCG was being released into the media (Figure 1). However, the indirect technique (*EEi*%) of quantifying the EGCG remaining in the supernatant after the encapsulation process indicated that about 20.8% (±5.85%) of EGCG was successfully loaded. The calculated agreement between the results of the two techniques for the loaded EGCG values was over 95% (*t*-test *p* = 0.634, *n* = 6) and showed an average encapsulation efficiency (*EE_avg_*%) of 21.35% (±4.6%), as shown in Figure 2.

### 3.2. EGCG Release Analysis Using UV-Vis

The mean and standard deviation (SD) of the accumulated release of EGCG up to 8 weeks are summarized in Table 2. The amount of EGCG released over time was compared across the adhesive groups containing EGCG (G3 and G4). The analysis revealed a statistically significant reduction in the amount of EGCG released (μg) from G3 at every time point (*p* < 0.05). The highest difference in the amount of EGCG released was observed on day 1 between the G3 (M = 1.07 μg, SD = 0.24) and G4 (M = 2.85 μg, SD = 0.14) groups; t(4) = −11.238, *p* < 0.001. The 56-day results showed a statistically significant difference in the accumulated release between the G3 (M = 3.71 μg, SD = 1.01) and G4 (M = 6.18 μg, SD = 0.76) adhesive groups; t(4) = −3.391, *p* = 0.028. The results indicated that HNTs played a positive role in slowing down the EGCG release.

### 3.3. MMP-Mediated β-Casein Cleavage Assays

**Figure 3:**
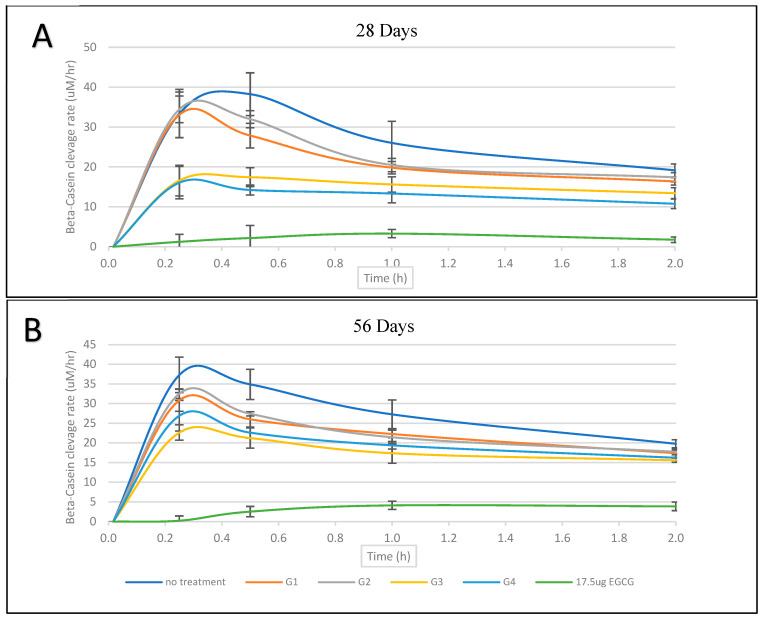
β-casein substrate (12.5 mg/mL) cleavage rate by MMP-9 (0.1 μg/m) at 28 days (**A**) and 56 days (**B**).

The bCN cleavage ratio (% relative to the baseline) and cleavage rates (μM/h) results are summarized in Table 3. Comparative results for the cleavage rates (μM/h) between 28 and 56 days are illustrated in Figure 3 and show a substrate-limiting effect after a 0.25 h time point.

The statistical analyses revealed significant differences between the groups in the bCN cleavage ratios (% relative to baseline) after 28 days (F(5,12) = 55.82, *p* < 0.001) and 56 days (F(5,12) = 90.34, *p* < 0.001). Tukey’s post hoc tests for multiple comparisons found that the mean value of bCN cleavage ratios for the 28-day samples was significantly higher in the negative control group (no treatment) compared to G3 (*p* = 0.005, 95% CI = [6.64, 38.05]) and G4 (*p* < 0.001, 95% CI = [16.96, 48.38]), while there was no significant difference between the negative control group and G1 (*p* = 0.26) or G2 (*p* = 0.696). The analysis of the cleavage rates (μM/h) also indicated significant differences between the groups for the 28-day eluates (F(5,12) = 18.75, *p* < 0.001) and the 56-day eluates (F(5,12) = 40.852, *p* < 0.001). Tukey’s post hoc tests for multiple comparisons found that the cleavage rate (μM/h) of bCN was significantly faster in the negative control group compared to G3 (*p* = 0.022, 95% CI = [2.09, 31.52]) and G4 (*p* = 0.018, 95% CI = [2.61, 32.04]), while there was no significant difference found between the negative control group and G1 (*p* = 1.00) or G2 (*p* = 0.99). A similar statistical significance pattern was also found between the groups for the 56-day eluates.

**Figure 4:**
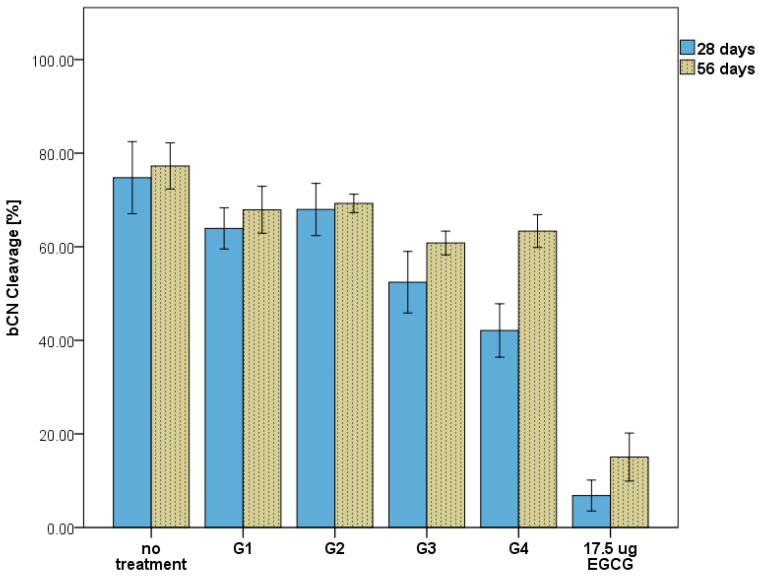
Total cleavage of bCN by MMP-9 detected by the intensity band relative to the baseline.

Comparing the results between 28 and 56 days within the group showed, in general, a noticeable increase in the MMP-9 cleavage activity (Figure 4). However, this difference was not statistically significant except for the G4 (0.15%EGCG) eluates, which at 56 days (63.35% ± 3.52) were shown to be significantly higher compared to those at 28 days (42.09% ± 5.71); *t*(4) = 5.5, *p* = 0.01. In comparison, no significant difference was found in G3 (0.15% EGCG-7.5% HNT) for bCN cleavage activity between the two periods’ eluates; *t*(4) = 2.1, *p* = 0.15.

## 4. Discussion

This study aimed to examine the effect of EGCG and an EGCG-encapsulated HNT addition to dental adhesives’ ability to protect the collagen fibrils at the hybrid layer against degradation by proteolytic activity after restoration placement. The results of this study provide meaningful insights into the amount of EGCG that can be loaded into HNTs, the amount of EGCG that is released from the adhesive, and the ability of the EGCG in the eluates to retain its inhibitory effects. Indeed, the results indicated that the released EGCG showed a dose-dependent inhibition of MMP-9, while the HNTs helped slow down the release rate without reducing the benefits of the EGCG addition.

The concentration of EGCG in this study was 1.6 mg/mL (3.5 mM); as such, it may be considered high compared to previous studies that involved modifying the adhesive with EGCG as they ranged between 0.025 and 2.18 mM [21,22,23,24]. However, these prior studies experimented with dental adhesives in different physical, chemical, biological, and mechanical testing settings. The conclusions reported in these studies, in general, indicated an improved or no difference between the control group and experimental groups in terms of bonding strength, flexural strength, degree of conversion, and antibacterial effect, except for one study that reported that EGCG had adverse effects on a commercially available dental adhesive called the Filtek Silorane System [23].

Our study utilized UV-vis spectrometry to detect and quantify EGCG particles and determine HNTs’ potential to encapsulate EGCG for drug release. The linearity and working range of the UV-vis method from unpublished data were found adequate for detecting EGCG to evaluate the encapsulation efficiency and drug release. UV-vis measurements of EGCG in water and ethanol solvents demonstrated linearity exceeding 99% with a working range between 0.02 μg/mL and 40 μg/mL. The validation of the UV-vis method in detecting EGCG and other tea catechins in terms of specificity, linearity, quantification limits, precision, and robustness has already been verified by multiple studies [29,31,32].

The encapsulation efficiency analyses revealed that the HNT could hold 21.35% (±4.6%) of the EGCG, and it was used in approximating the equivalent EGCG quantity in the HNT-free EGCG group (G4). Palasuk et al. (2018) showed a comparable drug loading capacity for HNTs and Doxycycline ranging between 19 and 38% [33]. Veerabadran et al. (2007) studied the interactions between HNTs and Dexamethasone, Furosemide, and Nifedipines and found a maximum drug loading of 12% [34]. The differences in HNT drug loading capacity between studies can be attributed to variations in the HNT source, drug chemistry, and the testing techniques involved.

The accumulative EGCG release data collected by UV-vis showed that 7.5%*w*/*v* of HNTs reduced the EGCG release by 52% compared to the EGCG specimen without HNTs. Since the volume of a specimen and the loaded EGCG amount were identified, it was possible to estimate how much EGCG remained at each point of the release detection. After 56 days, the EGCG-only adhesive groups released about 10% of their EGCG capacity, while the EGCG-HNT groups released about 5%. However, the physical and chemical properties of the solvent, such as temperature and acidity, can significantly impact release rates. Therefore, the study was limited since it only used purified water at room temperature for evaluating the EGCG release and detection.

Both gelatin and casein zymography have been widely used to study the MMP expression and MMP inhibitors’ potent activities [35,36,37]. β-casein (bCN) is a proline-rich protein that belongs to the most dominant protein family found in dairy, which is also widely used as a substrate for detecting several MMPs, with a preferential detection potential for MMP-1, MMP-7, MMP-9, MMP-12, and MMP-13 [38,39]. The β-casein cleavage method was selected for the current study due to its relatively low molecular weight (29 kDa) and ability to migrate in the gel during electrophoresis to facilitate the detection of MMP-9 and EGCG inhibitory effects.

The release analysis revealed that higher levels of EGCG were present in the eluates from HNT-free adhesive groups than in the EGCG-encapsulated HNT groups. However, the MMP inhibition results, especially those observed after 56 days, did not reflect the projected effects, as both HNT-EGCG and EGCG adhesives exhibited comparable results in terms of MMP-9 inhibition. At 28 days, G3 and G4 showed similar bCN cleavage rates, but the gap was increased at 56 days, which may be contributed to the higher amount of EGCG released in G3. Additionally, the positive control group that contained a reference baseline EGCG (17.5 μg) showed an increase in the bCN cleavage by MMP-9 (6.83% at 28 days and 15.04% at 56 days), with the “storage time” being the only variable that was implemented in that group. One speculation of this outcome can point to the possibility of EGCG undergoing oxidative degradation after release, thereby decreasing its bioavailability [40]. Indeed, several studies have shown that green tea catechins, such as EGCG, are susceptible to degradation and loss of their biological activity when diluted in an aqueous solution over time [41,42,43]. Oxygen, pH, and temperature have been identified as factors that adversely affect EGCG’s native chemical state and physiological activity [44]. Consequently, further research is required to evaluate the MMP inhibitory effect of the EGCG when exposed to oral fluids under physiological and pathological conditions.

In conclusion, within the current study’s limitations, the use of HNT encapsulation to modify a dental adhesive was found to slow the rate at which EGCG was released without significantly interfering with the purpose of the EGCG addition as an MMP inhibitor. Further in vitro and in vivo tests are needed to confirm the EGCG-encapsulated adhesive’s potential to improve bonding durability and the role it can play in extending the life span of restorations.

## Figures and Tables

**Table 1:** Summary of the adhesive groups with the ratios of the components and the final EGCG concentrations.

	Adhesive Group	Adhesive (mL)	HNT (mg)	EGCG (mg)	Final EGCG Concentration (mg/mL)
G1	Control adhesive	5	-	-	0
G2	7.5% HNT	5	375	-	0
G3	7.5% HNT-0.15% EGCG	5	375	37.5 mg (~8.0 mg *)	1.6
G4	0.15% EGCG	5	-	8.0 mg	1.6

* Successfully loaded into HNTs determined by the drug loading efficiency results (~21.35% loaded).

**Table 2:** EGCG accumulative release (μg) detected by UV-vis from EGCG-containing adhesive specimen eluates.

Group		1 Day	7 Days	14 Days	28 Days	56 Days
G3 7.5% HNT-0.15% EGCG	Mean	1.07 ^a^	1.63 ^a^	2.23 ^a^	2.95 ^a^	3.71 ^a^
	SD	0.24	0.36	0.51	0.88	1.01
G4 0.15% EGCG	Mean	2.85 ^b^	3.97 ^b^	4.77 ^b^	5.54 ^b^	6.18 ^b^
	SD	0.14	0.14	0.12	0.31	0.76

Different letters indicate statistically significant differences (*p* < 0.05) within the column.

**Table 3:** bCN cleavage by MMP-9 (% relative to the baseline) and the cleavage rate (μM/h) results.

Group	bCN Cleavage % (SD)		bCN Cleavage Rate (μM/h) (SD)	
	28 Days	56 Days	28 Days	56 Days
blank	74.76 (7.70) ^a^	77.26 (4.94) ^a^	33.40 (7.43) ^a^	37.29 (5.55) ^a^
G1 (Control adhesive)	63.92 (4.40) ^a,b^	67.90 (5.00) ^a,b^	33.11 (7.00) ^a^	30.83 (3.42) ^a,b,c^
G2 (7.5% HNT)	67.96 (5.59) ^a,b^	69.26 (1.96) ^a,b^	34.45 (4.09) ^a^	32.52 (1.48) ^a,b^
G3 (7.5% HNT-EGCG)	52.42 (6.57) ^b,c^	60.80 (2.54) ^b^	16.59 (4.68) ^b^	21.64 (2.63) ^c^
G4 (0.15% EGCG)	42.09 (5.71) ^c^	63.35 (3.52) ^b^	16.07 (4.99) ^b^	26.92 (4.75) ^b,c^
17.5 ug EGCG	6.83 (3.30) ^d^	15.04 (5.13) ^c^	1.30 (2.24) ^c^	1.71 (2.63) ^d^

Different letters indicate statistically significant differences at 0.05 level.

## Data Availability

Not applicable.
